# A High Docosahexaenoic Acid Diet Alters the Lung Inflammatory Response to Acute Dust Exposure

**DOI:** 10.3390/nu12082334

**Published:** 2020-08-04

**Authors:** Edward C. Dominguez, Art J. Heires, Jacqueline Pavlik, Tricia D. Larsen, Stephanie Guardado, Joseph H. Sisson, Michelle L. Baack, Debra J. Romberger, Tara M. Nordgren

**Affiliations:** 1 Division of Biomedical Sciences, School of Medicine, University of California Riverside, Riverside, CA 92521, USA; edomi007@ucr.edu (E.C.D.); Eguar003@ucr.edu (S.G.); 2 Pulmonary, Critical Care, Sleep and Allergy Division, University of Nebraska Medical Center, Omaha, NE 68198, USA; aheires@unmc.edu (A.J.H.); jpavlik@unmc.edu (J.P.); jsisson@unmc.edu (J.H.S.); dromberg@unmc.edu (D.J.R.); 3 Environmental Influences on Health and Disease Group, Sanford Research, Sioux Falls, SD 57104, USA; Tricia.Larsen@SanfordHealth.org (T.D.L.); Michelle.Baack@SanfordHealth.org (M.L.B.); 4 Department of Pediatrics, University of South Dakota—Sanford School of Medicine, Sioux Falls, SD 57104, USA; 5 VA Nebraska-Western Iowa Healthcare System, Omaha, NE 68105, USA

**Keywords:** omega-3 fatty acids, docosahexaenoic acid (DHA), organic dust, lung inflammation, specialized pro-resolving mediators (SPM)

## Abstract

Agricultural workers are at risk for the development of acute and chronic lung diseases due to their exposure to organic agricultural dusts. A diet intervention using the omega-3 fatty acid docosahexaenoic acid (DHA) has been shown to be an effective therapeutic approach for alleviating a dust-induced inflammatory response. We thus hypothesized a high-DHA diet would alter the dust-induced inflammatory response through the increased production of specialized pro-resolving mediators (SPMs). Mice were pre-treated with a DHA-rich diet 4 weeks before being intranasally challenged with a single dose of an extract made from dust collected from a concentrated swine feeding operation (HDE). This omega-3-fatty-acid-rich diet led to reduced arachidonic acid levels in the blood, enhanced macrophage recruitment, and increased the production of the DHA-derived SPM Resolvin D1 (RvD1) in the lung following HDE exposure. An assessment of transcript-level changes in the immune response demonstrated significant differences in immune pathway activation and alterations of numerous macrophage-associated genes among HDE-challenged mice fed a high DHA diet. Our data indicate that consuming a DHA-rich diet leads to the enhanced production of SPMs during an acute inflammatory challenge to dust, supporting a role for dietary DHA supplementation as a potential therapeutic strategy for reducing dust-induced lung inflammation.

## 1. Introduction

Exposure to agricultural organic dusts can lead to the development of acute and chronic lung diseases including asthma, bronchitis, and chronic obstructive pulmonary disease (COPD) in exposed individuals [1,2,3,4]. Agricultural workers regularly exposed to these environmental pollutants are at increased risk of developing respiratory diseases [5,6,7,8]. Organic dusts from animal confinement facilities have previously been reported to contain a large variety of bacterial components, proteases, and particulates that induce a pro-inflammatory response following exposure [9,10,11]. Specifically, acute dust exposure from swine confinement facilities has been shown to stimulate the release of pro-inflammatory cytokines such as TNF-α and IL-6 [11,12]. There are several well-established preventative approaches to help minimize the exposure risk for these individuals, including the use of respirators or ventilator masks. Unfortunately, compliance with the prescribed use of personal protective equipment is not routinely followed by the farming community [13,14].

A multitude of proteins, mediators, and biological processes aid in the resolution of the inflammatory process, including the metabolism of omega-3 polyunsaturated fatty acids (omega-3 PUFAs) into specialized pro-resolving mediators (SPMs) [15,16]. Research has shown that a traditional Western diet is comprised of about 15:1 omega-6 to omega-3 fatty acids as opposed to the ideal 1:1 ratio [17]. Both omega-3 and omega-6 PUFAs are metabolized by the same enzymes to form bioactive lipids that are primarily SPMs or pro-inflammatory lipid mediators, respectively [18,19,20,21,22]. The omega-3 PUFA docosahexaenoic acid (DHA) is metabolized by lipoxygenase, cyclooxygenase, and epoxygenase enzymes into various SPMs, such as resolvins and maresins, which reduce inflammation in the lung [19,23,24,25,26]. During the inflammatory process, these SPMs function as anti-inflammatory and pro-resolving mediators by regulating various processes including neutrophil and macrophage influx into the lung, and promoting tissue repair and immunity [27,28]. Our previous investigations have examined the effectiveness of short-term 7-day DHA supplementation in altering the lung inflammatory response to organic dust exposure, where we identified DHA-mediated reductions in the lung inflammatory response and specific alterations to the airway epithelium following acute exposure to extracts of organic agricultural dusts collected from hog confinement facilities (HDE) [29]. No significant alterations in the DHA-derived SPM resolvin D1 (RvD1) were found upon the lavage of DHA-supplemented mice. To build on this study, we sought to test whether increasing the duration of dietary DHA treatment would lead to alterations in endogenous SPM production during an acute inflammatory challenge with environmental dust. Specifically, we tested whether a 4-week DHA-supplemented diet regimen prior to exposing mice to agricultural dust collected from a hog confinement facility would promote the enhanced production of SPM and an altered inflammatory response of dust-induced inflammation. To do so, we used a well-established mouse model using HDE to induce acute lung inflammation [10,30,31,32]. Following exposure, we assessed the lung’s immune response to dust exposure, including the NanoString gene expression profiling of 561 genes related to the immune response, in addition to assessing alterations in SPM production in DHA-diet- versus control (no DHA)-diet-fed mice. Overall, our findings demonstrate that a high DHA diet can enhance the biosynthesis of SPM during acute lung inflammation caused by organic dust exposure and alter the transcript-level gene expression changes of pro-/anti-inflammatory genes.

## 2. Materials and Methods

### 2.1. Reagents

Murine tumor necrosis factor-α (TNF-α), interleukin-6 (IL-6), chemokine C-X-C motif ligand 1 (CXCL1), amphiregulin (AREG), granulocyte-macrophage colony-stimulating factor (GM-CSF), myeloperoxidase (MPO), monocyte chemoattractant protein-1 (MCP-1), and macrophage migration inhibitory factor (MIF), Duoset ELISA kits were purchased from R&D Systems (Minneapolis, MN, USA). RvD1 and RvD2 murine ELISA kits were purchased from Cayman Chemicals (Ann Arbor, MI, USA). DHA and standard mouse chow diets were prepared by Envigo (Madison, WI, USA) as previously described [30].

### 2.2. Preparation of Organic Dust Extract

Organic agricultural dusts collected from hog confinement facilities were prepared into extracts (HDE) as previously described [30]. In short, settled dusts from swine confinement facilities were collected from surfaces 1 m above the ground and suspended in Hank’s Balanced Salt Solution at a 100 mg/mL concentration. The mixture was centrifuged and filtered at 0.22 μm to form the 100% HDE extract, which was stored at −20 °C for future use and later diluted to a 12.5% HDE (vol/vol) concentration for animal studies, using sterile phosphate-buffered saline (PBS). The characterization of the bacterial components in the dust has been previously documented [9].

### 2.3. Animal Care

Male wildtype C57BL/6J mice at 5–8 weeks of age were obtained from The Jackson Laboratory (Bar Harbor, ME, USA) and housed in the University of Nebraska Medical Center (UNMC) Comparative Medicine Facilities. The mice were allowed free access to food and water and housed in micro-isolator cages (five per cage). The food was changed weekly by the investigators, while the water was changed by UNMC animal care staff. Each mouse was weighed weekly (see Figure S1 for initial and final weights) and examined for any signs of distress. All related experiments and procedures were approved by the UNMC Institutional Animal Care and Use Committee.

### 2.4. In Vivo Model of Dust Exposure

We employed a previously established murine model that utilizes an intranasal exposure to HDE to induce airway inflammation for our in vivo investigations [30,31]. The studies were performed as 3 independent experimental sets, for total of 8 mice for the saline-treated groups and 12 mice for the HDE-treated groups. The mice were lightly anesthetized by isoflurane inhalation prior to receiving a single intranasal challenge of 50 μL of sterile saline (PBS) or 12.5% HDE. Four weeks prior to receiving the intranasal instillation, the mice were initiated on either a DHA-rich diet or control diet containing no DHA. The animal chow diets were prepared by Envigo (Madison, WI, USA) using a base AIN-93G diet. The DHA diet preparation was the same as that previously described [30,33]. Briefly, to prepare the DHA-rich diet, soybean oil from the AIN-93G base diet was replaced with DHASCO oil (DSM Nutritional Products, Kingstree, SC, USA), a DHA-based oil containing 39.2% DHA and high-oleic safflower oil. The control diet, containing no DHA, modified the same AIN-93G diet by replacing soybean oil with high-oleic safflower oil. The newly formulated DHA-rich diet provides the mice with approximately 1.4% of their total caloric intake strictly from DHA. Five hours following the single HDE intranasal challenge, the mice were euthanized. Blood was collected, bronchoalveolar lavage fluid (BALF) retrieval was performed using three 1 mL washes with sterile PBS, and the left lungs were collected and stored in RNAlater (Thermo Fisher, Waltham, MA, USA). BALF from the first lavage was stored and used to measure cytokine and chemokine levels by ELISA. Total cell counts from the BALF were obtained, and differential cell counts were determined microscopically on cytocentrifuge slides (Cytopro, ELITechGroup, Logan, UT, USA), which were stained using DiffQuick (Siemens, Newark, DE, USA).

### 2.5. Neutrophil Extracellular Trap (NET) Scoring

Cytospins were prepared from the BALF of each mouse following the administration of the diets and exposures. The neutrophil extracellular trap (NET) scoring of the BALF cytospins stained with the Diffquick Hema 3 Stat Pack (Fisherbrand, Pittsburg, PA) was performed as previously described [34]. In short, the entire periphery of each cytospin was divided into twenty-seven regions as this is where NETs are concentrated, and each region was assigned a NET score of 0 (no NETs), 1 (rare NETs), 2 (moderate NETs), or 3 (widely distributed NETs). All 40 cytospins from the mice used across the three separate experiments were assessed for NETs.

### 2.6. Analysis of Cytokines and Chemokines

Cytokine and chemokine levels were measured from the cell-free BALF of the first PBS wash using murine-specific ELISAs. We examined the levels of TNF-α, IL-6, CXCL1, AREG, GM-CSF, MPO, MCP-1, and MIF using commercially available kits (*Duoset* ELISA development kits, R&D Systems) as well as RvD1 and RvD2 (Cayman Chemical) according to the manufacturer’s instructions.

### 2.7. Fatty Acid Blood Levels

Whole blood was collected from the axillary artery and placed in BD Microtainer Tubes (Becton Dickinson, Franklin Lakes, NJK). Fatty acid analysis was performed by direct transesterification using 14% boron-trifluoride methanol and hexane solution with an internal standard (17:0 heptadecanoic acid) at 100 °C for 60 min. After cooling, HPLC-grade water was added and the sample was vortexed and centrifuged for phase separation. Fatty acid methyl-esters in the hexane phase were analyzed by capillary gas chromatography (GC) on an Agilent 7890A GC (Agilent Technologies, Santa Clara, CA, USA) equipped with an Agilent CP7489 Capillary Column (100 m length × 0.25 mm internal diameter × 0.36 mm outer diameter, 0.20 μm film thickness). Hydrogen was the carrier gas. Fatty acids of carbon length 10 to 24 were detected with a flame ionization detector and recorded using the ChemStation interface system (Agilent Technologies, Santa Clara, CA, USA). Peaks were identified using the Open LAB Chromatography Data System and corresponding authentic standard, 37 FAME mix (Sigma Aldrich, St. Louis, MO, USA). The whole blood composition (wt/wt% of the sample) is reported for individual FAs. The individuals performing the analyses were blinded to the study groups.

### 2.8. NanoString Gene Expression

Randomly selected left lung tissues (*n* = 24) out of the possible 40 mice, representing tissues collected across three unique experimental trials, were homogenized and RNA was extracted using the PureLink RNA Mini Kit (Invitrogen, Carlsbad, California, USA). RNA sample quality was quantified using the NanoDrop ND-100 (NanoDrop Technologies, Inc, Wilmington, DE, USA) and an Agilent 2100 Bioanalyzer (UC Riverside Core Facilities, Agilent Technologies, Santa Clara, CA, USA). The assessment of transcript-level gene expression changes was performed using the NanoString mouse Immunology Panel (NanoString Technologies, Seattle, WA, USA), a codeset designed to target 561 genes related to inflammation and the immune response. Fifty to one hundred nanograms of total RNA was mixed with the codeset and reporter probes and hybridized for 16 h to form a purified target–probe complex that was then imaged and quantified with a nCounter Sprint profiler. Gene expression data analysis was performed using the nCounter Analysis System, nSolver 4.0 software. The expression data were normalized by using the geometric mean of 4 housekeeping genes: *OAZ1, PPIA, RPL19,* and *EEF1G*. Following normalization, 20 of the samples passed normalization and were viable to use for the subsequent analyses, including 4 control diet + saline, 6 control diet + HDE, 3 DHA diet + saline, and 7 DHA + HDE samples. Raw and normalized NanoString data are deposited at https://www.ncbi.nlm.nih.gov/geo/query/acc.cgi?acc=GSE155539. Following initial gene expression analysis, the STRING database was used to evaluate protein–protein interactions amongst high- or low-expressing genes.

### 2.9. Statistical Analyses

Statistical analysis and graphing were performed using the Prism Software by GraphPad (San Diego, CA, USA). All reported data are presented as the mean ± standard error. Statistical calculations were performed using two-way ANOVA with Tukey’s post hoc test for multiple comparisons within the groups, and significance was set at *p* ≤ 0.05. For graphical presentation, the # symbol is used to indicate significant differences in post hoc comparisons between the DHA and control diet groups. The * symbol is used to indicate significant differences in the post hoc comparisons between saline- and HDE-treated mice within diet groups. The + symbol is used to indicate significant interactions between diet and treatment groups.

Sample quality control for the NanoString gene expression dataset was performed manually by eliminating probes with fewer than 82 counts from the data set. This was executed by performing a background subtraction (mean +/− two standard deviations of negative controls) to more efficiently differentiate between biological or technical variation amongst the samples. Raw *p*-values from the differential expression analyses were used to assess gene expression data. All heat maps and data cluster sets were produced using the nCounter Analysis and Advanced Analysis packages in nSolver 4.0 (NanoString Technologies, Seattle, WA, USA).

## 3. Results

### 3.1. A High-DHA Diet Alters Blood Omega-3 and Omega-6 PUFA Levels

**Figure 1:**
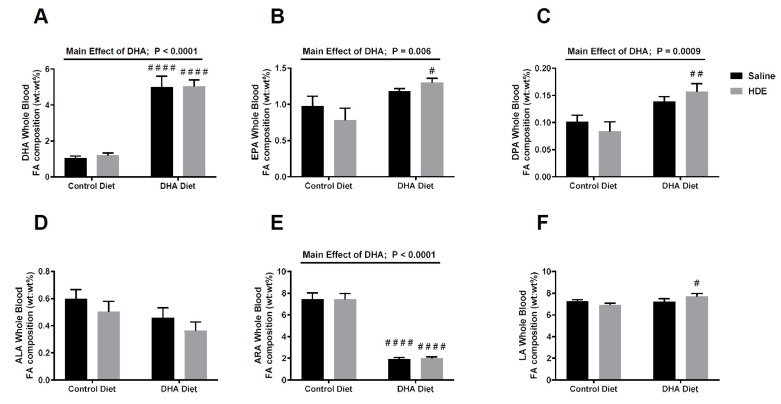
Fatty acid blood levels collected from the docosahexaenoic acid (DHA)- and control-diet-fed mice 5 h after the instillation of HDE or saline. Fatty acid blood levels of n-3 PUFA DHA (**A**), eicosapentaenoic acid (EPA) (**B**), docosapentaenoic acid (DPA) (**C**), alpha-linolenic acid (ALA) (**D**) and n-6 PUFA arachidonic acid (ARA) (**E**) and linoleic acid (LA) (**F**), measured in mice following administration of a diet and exposure. Significance for the main effect of the DHA diet is shown with the *p*-values and significance bars on top of the DHA, EPA, DPA, and ARA graphs. Tukey post hoc comparisons between the DHA- and control-diet-treated conditions are annotated with the # symbol. (# *p* < 0.05; ## *p* < 0.01; #### *p* < 0.0001).

Following euthanasia at 5 h post-HDE challenge, whole blood was isolated and used to assess the levels of various medium- and long-chain fatty acids (C10–24). Mice that were given a high-DHA diet showed significantly increased levels of the omega-3 PUFAs DHA (Figure 1A, *p* < 0.0001), eicosapentaenoic acid (EPA; Figure 1B, *p* = 0.006), and docosapentaenoic acid (DPA; Figure 1C, *p* = 0.0009). Conversely, DHA-diet-fed mice exhibited significantly decreased levels of the omega-6 PUFA arachidonic acid (ARA; Figure 1E, *p* < 0.0001). There was no significant main effect for either the omega-3 PUFA alpha linolenic acid (ALA; Figure 1D, *p* = 0.067) or omega-6 PUFA linoleic acid (LA; Figure 1F, *p* = 0.076), as both slightly missed significance. There was, however, a significant difference in the LA levels for the control-diet- versus DHA-diet-fed mice challenged with HDE (LA; Figure 1F).

The sums of the omega-6 PUFAs (ARA and LA) and omega-3 PUFAs (EPA, DPA, DHA, and ALA) were calculated to identify the ratio of omega-6 to omega-3 PUFAs for the mice that were fed a control diet compared to the DHA diet, as shown in Table 1. Mice fed a DHA-rich diet had an approximately 2.3:1 ratio of omega-6 PUFAs (ARA and LA) to omega-3 PUFA (EPA, DPA, DHA, and ALA), compared to those fed a control diet, which had an approximately 6.8:1 ratio.

### 3.2. A High-DHA Diet Impacts Overall Lung Cellular Influx in Mice Following Acute HDE Exposure

**Figure 2:**
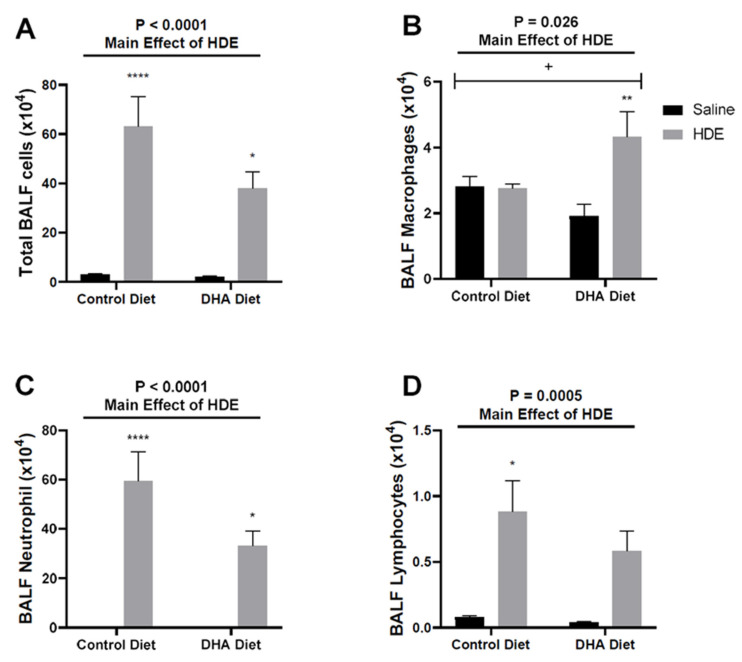
Effects of a high-DHA diet on total and immune cell lung influx after HDE instillation. Control- or DHA-diet-fed mice were challenged with a single intranasal HDE exposure. Five hours following exposure, bronchoalveolar lavage fluid (BALF) was collected and assessed for cellular influx including total BALF cells (**A**), BALF macrophages (**B**), BALF neutrophils (**C**), and BALF lymphocytes (**D**). The main effects of HDE vs. saline are indicated by the top lines with corresponding *p*-values. The * symbol above the HDE bars represents the statistical significance of the difference between the HDE- and saline-treated conditions within the same diet group (* *p* < 0.05; ** *p* < 0.01; **** *p* < 0.0001). Significant interactions between diet and exposure are depicted by the + symbol (+ *p* < 0.05).

In our 7-day DHA oral gavage study, we reported that an acute (single) HDE challenge elicited a potent lung inflammatory response [29]. Additionally, we identified that a 7-day daily-gavage DHA pre-treatment prior to acute HDE challenge was effective in reducing the inflammatory effects of HDE [29,30]. To build on these initial findings, we opted to administer a DHA-rich diet to C57BL/6J mice 4 weeks prior to a single 50 µL intranasal challenge of 12.5% HDE to determine if a dietary intervention was similarly effective in reducing overall acute HDE-induced inflammation. Through these investigations, we identified that HDE exposure as a main effect was associated with significant increases in total BALF cellularity (Figure 2A), corresponding with significant increases in neutrophils (Figure 2C) and lymphocytes (Figure 2D). Of interest, we identified that HDE-exposed mice that were fed a DHA-rich diet displayed changes in the types of cells recruited; in DHA diet-fed mice, we identified a significant elevation of BALF macrophages following HDE exposure (Figure 2B) that was not evident in the control-diet-fed mice. Furthermore, we identified that neutrophil influx was decreased in DHA-diet-fed HDE-challenged mice compared to in control-diet-fed mice given HDE, although this missed statistical significance (*p* = 0.070; Figure 2C). We also identified a significant interaction (Figure 2B; *p* = 0.021) between diet and dust exposure for macrophage recruitment into the lung.

### 3.3. A DHA-Rich Diet Alters HDE-Induced Changes in Pro-Inflammatory Cytokine/Chemokine Release

**Figure 3:**
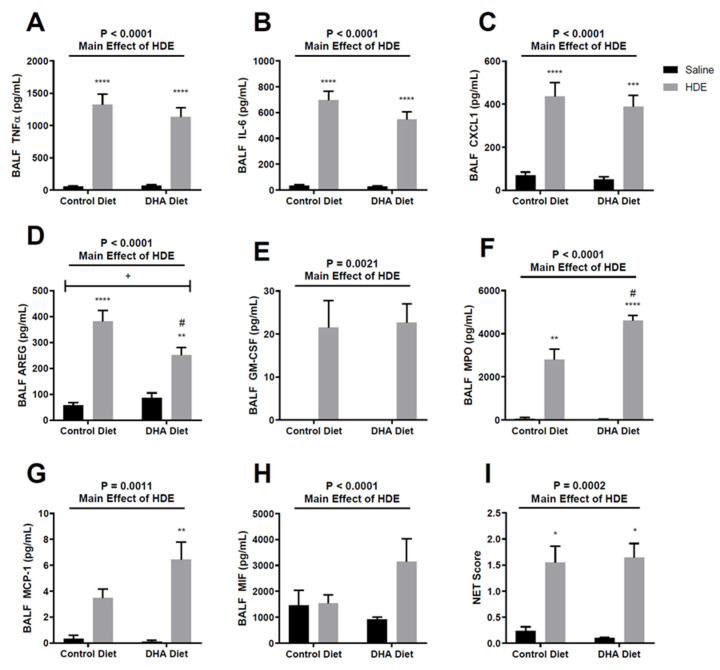
Effects of a DHA-rich diet on inflammatory mediator release and neutrophil extracellular trap (NET) formation following acute exposure to HDE. (**A**–**H**) BALF was collected from mice and screened for various cytokines and chemokines via ELISAs. (**I**) NET formation was assessed by scoring the BALF cytospins across all three experimental trials. The top line represents the main effect of HDE vs. saline with significance represented by *p*-values. Tukey post hoc comparisons are provided for DHA- vs. control-diet-treated conditions and are annotated by the # symbol (# *p* < 0.05). Significant differences between HDE- and saline-treated conditions within diet groups are represented by the * symbol above the HDE bars (* *p* < 0.05; ** *p* < 0.01; *** *p* < 0.001; **** *p* < 0.0001). Any significant interactions between diet and exposure are depicted by the + symbol (+ *p* < 0.05).

Acute HDE exposure leads to the production of pro-inflammatory cytokines and chemokines [12,29,30,35]. In our current investigation, we identified that acute HDE exposure led to increased levels of TNF-α, IL-6, CXCL1, AREG, GM-CSF, MPO, and MCP-1 (Figure 3) within the BALF, when compared to saline treatment. Mice that were administered a DHA-rich diet and exposed to HDE had significantly decreased levels of BALF AREG (Figure 3D, *p* = 0.017) and increased MPO levels (Figure 3F, *p* = 0.016) compared to HDE-instilled mice fed the control diet. There was also a significant interaction (Figure 3D; *p* = 0.021) between diet and dust exposure for AREG levels, with MPO missing significance (Figure 3F, *p* = 0.057) for this interaction. When assessing NET formation, we identified a significant major effect of HDE exposure on NET formation (Figure 3I) that was not impacted by diet.

### 3.4. A High-DHA Diet Is Associated with Increased Production of Resolvin D1

**Figure 4:**
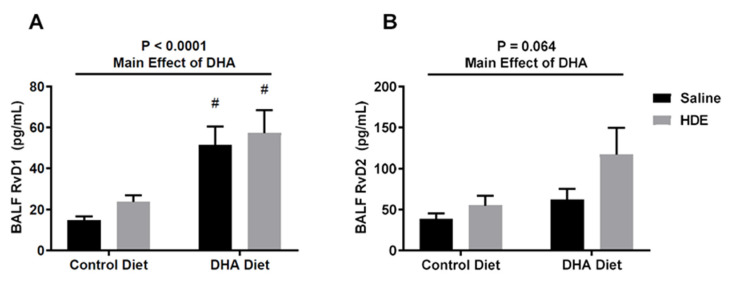
A high-DHA diet increases the production of Resolvin D1 in the lung following a single intranasal challenge with HDE. BALF levels of the specialized pro-resolving mediators (SPMs) Resolvin D1 (RvD1) (**A**) and Resolvin D2 (RvD2) (**B**) were assessed via ELISA following euthanasia. The main significance bars and *p*-values on the top represent the main effects of DHA on SPM production. Tukey post hoc comparisons for significant differences between the DHA- and control-diet-treated conditions are depicted by the # symbol (# *p* < 0.05).

To assess the impact of a longer-term DHA-rich diet on endogenous SPM production, we analyzed the BALF levels of DHA-derived resolvin D1 (RvD1) and Resolvin D2 (RvD2). DHA had an overall significant major effect on RvD1 production (Figure 4A), while RvD2 production slightly missed significance (Figure 4B). Here, DHA-diet-fed mice challenged with HDE exhibited significantly increased levels of the DHA-derived SPM RvD1 (Figure 4A, *p* = 0.011) in the BALF when compared to the control-diet-fed mice, with RvD2 levels also trending upward in the DHA-diet-fed mice but failing to reach significance (Figure 4B). Of note, RvD1 levels in DHA-diet-fed mice that were administered a saline intranasal challenge were also higher than those in control-diet-fed mice (Figure 4A, *p* = 0.027).

### 3.5. In Vivo Gene Expression Changes in DHA-Diet-Fed Mice Following Exposure to HDE

**Figure 5:**
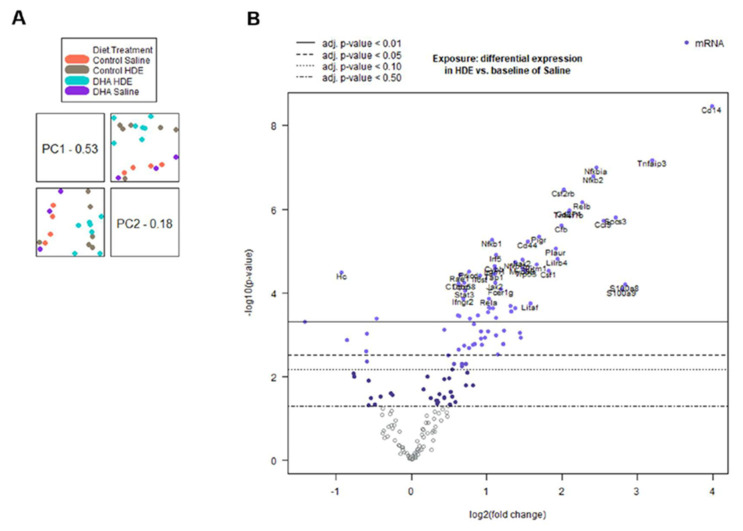
Principal component analysis and volcano plot of all 20 normalized samples. (**A**) Principal component analysis (PCA) and PCA scores displaying an overall strong clustering of related biological sample replicates for all 20 samples. (**B**) Volcano plot of expressed genes for the interaction between HDE and saline exposure among all 20 samples. *p*-value for volcano plot displayed as adjusted *p*-value.

To assess the transcript-level gene expression changes in the lungs of HDE-challenged mice fed a DHA-rich diet, a NanoString Mouse Immunology gene expression panel was used. Two separate advanced analyses were performed, one assessing the full 20-sample data set, and the other analysis assessing the 13 DHA vs. control-diet samples with exposure to HDE. All 20 NanoString samples were selected at random (see Materials and Methods), with 13 of them being HDE-exposed and seven, saline-exposed. Of the 13 HDE samples, six were control-diet-fed and seven were DHA-diet-fed. The full 20-sample data set showed expression changes for both the DHA diet and dust exposure, compared to the controls. After sample normalization, we performed the principal component analysis (PCA) of all 20 samples, which showed a clear clustering of exposure groups (Figure 5A). Corresponding with the PCA, the differential expression for the HDE vs. saline samples provided the greatest number of significantly altered genes of all the analysis sets (Figure 5B)

The numbers of statistically significant (*p* ≤ 0.05) genes that were differentially regulated of the 561 possible genes expressed are summarized in Table 2. As mentioned in the methods, genes below the 82-count threshold were removed from the analyses.

**Figure 6:**
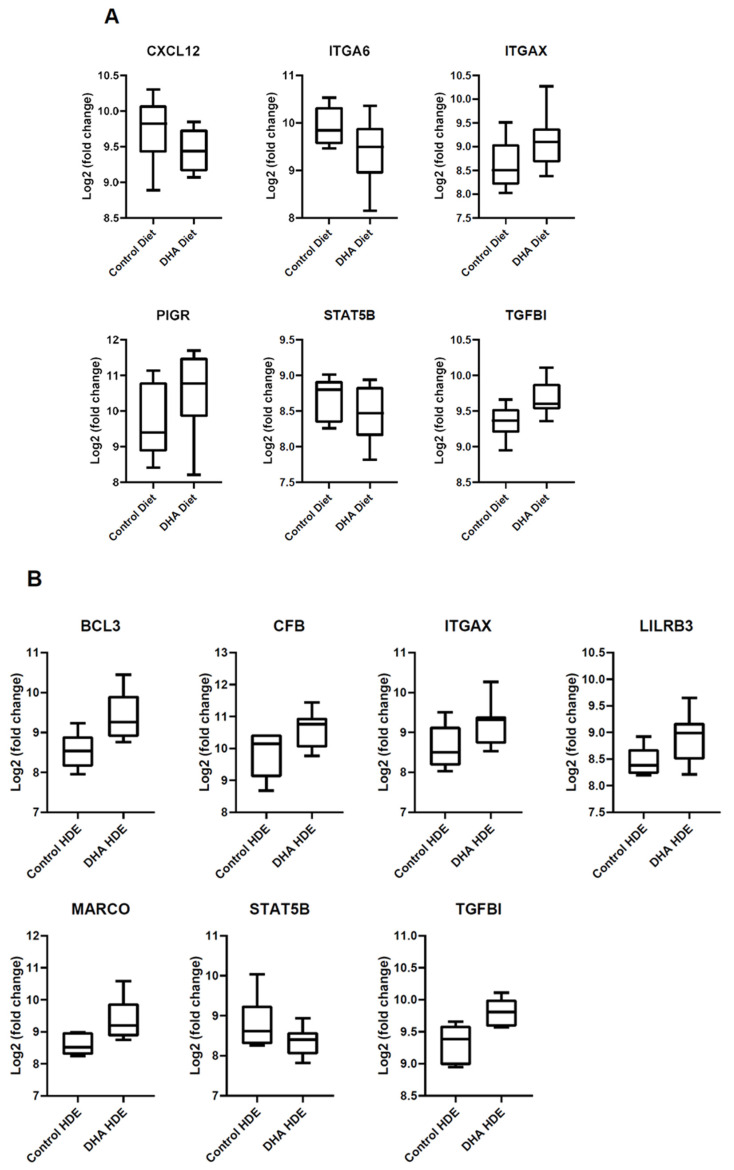
Box plots representing statistically significant (*p* ≤ 0.05) alterations in gene expression in HDE- or saline-challenged mice fed a high-DHA diet or control diet containing no DHA. Normalized Log2 values were exported from nSolver and plotted. (**A**) List of 6 genes that were significantly altered among all 20 DHA vs. control diet samples. (**B**) The 7 differentially regulated genes within the 13 DHA vs. control diet samples exposed to HDE.

Among the 20 DHA vs. control diet samples, *ITGAX*, *PIGR*, and *TGFBI* were significantly up-regulated, while *CXCL12*, *ITGA6*, and *STAT5B* were down-regulated (Figure 6A). Of the 110 significantly altered genes for the HDE vs. saline samples, we focused on the 20 most differentially expressed genes based on statistical significance (*p* ≤ 0.05). These 20 significantly altered genes were input into the STRING database and produced a significant protein–protein interaction (*p* < 1.0 × 10^−16^). Among the 13 DHA vs. control diet samples exposed to HDE, six genes were significantly up-regulated (*BCL3*, *CFB*, *ITGAX*, *LILRB3*, *MARCO*, and *TGFBI*) while *STAT5B* was down-regulated (Figure 6B). Three of these differentially expressed genes (*BCL3*, *LILRB3*, and *MARCO*) had counts below the 82-count threshold for all the saline-related samples, indicating minimal transcriptional expression under baseline conditions. As a result, these three genes were only assessed in the expression analysis of the DHA-vs.-control-diet, exposed-to-HDE sample set.

**Figure 7:**
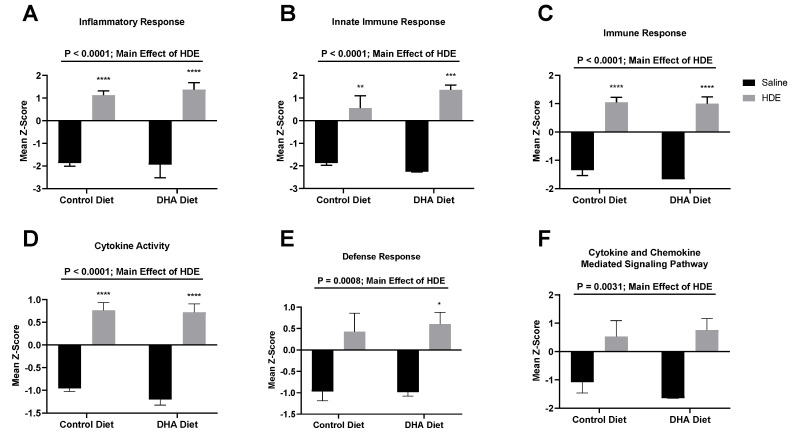
Expression changes in significantly altered pathways among all 20 DHA- vs. control-diet-fed mice. nSolver Advanced Analysis was performed to assess significant changes among pathway z-scores for various immunological pathways including inflammatory response (**A**); innate immune response (**B**); immune response (**C**); cytokine activity (**D**); defense response (**E**); and cytokine and chemokine-mediated signaling pathway (**F**). A major effect for HDE vs. saline exposure is indicated by the top significance bars and *p*-values. The * symbol above the HDE bars represents *p* < 0.05 versus all saline-treated conditions; ** *p* < 0.01; *** *p* < 0.001; **** *p* < 0.0001.

**Figure 8:**
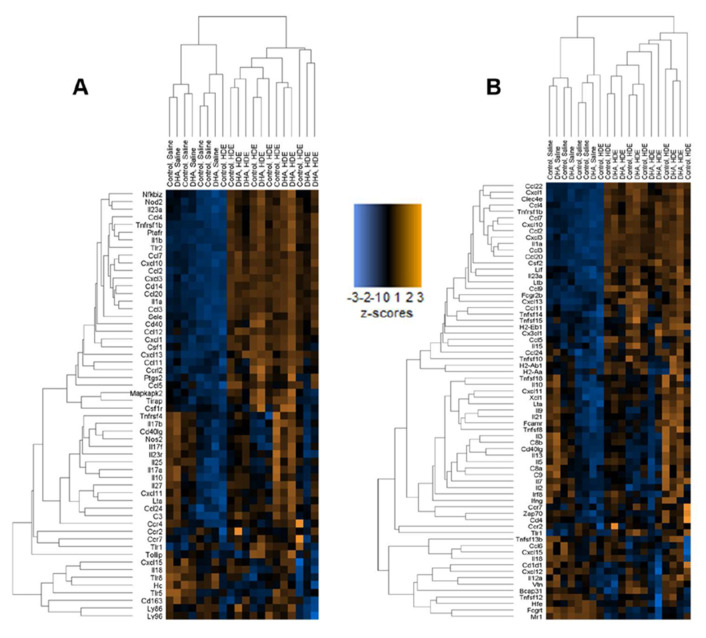
Hierarchal clustering shown by heat maps related to inflammation for all 20 normalized samples. (**A**) Heat map showing clustering of genes related to the inflammatory response. (**B**) Immune response heat map showing altered genes among all 20 samples.

The z-scores produced from the gene expression data were plotted and analyzed by two-way ANOVA to identify significant pathway alterations between the diet and exposure experimental groups for genes related to immunological pathways. For genes related to the inflammatory response, the immune response, the innate immune response, cytokine activity, the defense response, and cytokine- and chemokine-mediated signaling pathways, HDE exposure was associated with a significant up-regulation of genes related to these responses when compared to saline exposure (Figure 7 and Figure 8). A brief examination of the sole effect of HDE vs. saline for additional altered pathways revealed significant changes in the activation of pathways relating to the cell surface, receptor activity, extracellular region, plasma membrane, and signal transduction.

## 4. Discussion

Acute and chronic dust exposure can lead to a variety of lung inflammatory diseases among exposed individuals, especially agricultural workers [1,2,3,36]. The use of omega-3 PUFAs and their SPM metabolites as a therapeutic approach to reduce dust-induced lung inflammation shows promise in reducing the lung’s inflammatory response to environmental dust exposure [18,30,35]. We have previously shown that a single HDE exposure stimulates a strong inflammatory response that includes the release of pro-inflammatory cytokines and an increased neutrophil influx in the BALF of exposed mice [31]. In this study, we supplemented a standard mouse diet with DHA to assess the impact of a high-omega-3-PUFA diet as a preventative approach to mitigate the risk of inadvertent exposure by altering the lung inflammatory response. Through these investigations, we found that pretreating mice with a DHA-rich diet led to significant changes in the lung’s inflammatory response to acute dust exposure. Unlike control-diet-fed mice, we identified that DHA-diet-fed mice exhibited a significant increase in BALF macrophages following HDE challenge (Figure 2C) along with a significant increase in BALF SPM RvD1 (Figure 4A). Furthermore, we identified alterations to AREG and MPO release in control-diet-fed versus DHA-diet-fed animals exposed to HDE, and significant alterations in numerous immune-associated genes. Together, these data indicate that a high-DHA diet leads to significant alterations in the lung’s immune response to acute agricultural dust exposure, warranting its continued exploration as a potential modifier of the pathological inflammation experienced by individuals living or working in agricultural settings.

An omega-6 fatty acid to omega-3 fatty acid ratio of ~ 1:1 is thought to be associated with protective health effects by promoting the resolution of inflammation [17,23]. The dietary consumption of supplements high in omega-3 PUFAs such as fish oil has been shown to be effective in reducing the risk of cardiovascular and lung diseases [37,38,39]. In this study, mice fed a DHA-rich diet had a roughly three times lower omega-6 to omega-3 PUFA ratio than those on a control diet, suggesting that the employment of a high-DHA diet effectively increases the overall blood levels of omega-3 PUFAs. A lower dietary intake of omega-3 PUFAs in conjunction with the high degree of dust exposure agricultural workers experience can amplify their risk of developing acute/chronic lung diseases. A recent investigation identified an omega-6 PUFA/omega-3 PUFA ratio of over 50:1 in a cohort of COPD patients with agricultural work histories, highlighting the opportunity for impact in this population [40]. It is worth noting that even in our control-diet-fed mice, we identified an omega-6/omega-3 PUFA ratio of approximately 6.8:1, which is substantially lower than the typical Western diet ratio [17], suggesting more pronounced responses might have been identified in a dietary supplementation scheme yielding a higher omega-6/omega-3 PUFA ratio in control-diet-fed mice. In addition, while we identified significant elevations in the DHA-derived RvD1 in our DHA-diet-fed mice, a limitation to our studies is that we did not assess omega-6-PUFA-derived mediators. Doing so would have allowed for a more complete assessment of the ramifications of decreasing the omega-6 fatty acid to omega-3 fatty acid ratio for modifying bioactive lipid generation.

Macrophages are known mediators of both inflammation and resolution/repair, and are both responsive to SPM and also generators of SPM during an inflammatory response in the lung [41,42]. In our previous 7-day DHA oral supplementation study, we found no statistical differences for macrophage influx or SPM production in the lungs of DHA-treated mice compared to the controls following HDE exposure [29]. We did, however, see that DHA reduced HDE-elicited responses in the immortalized monocyte cell line THP-1 [29]. The increased macrophage influx into the lungs along with the significant interaction between diet and dust exposure that we identified in these current investigations is unique to this dietary intervention method and suggests that a high-DHA diet may alter macrophage influx or activation kinetics, possibly in part through the increased generation of the SPM RvD1. RvD1 is a member of the resolvins family of SPMs that are produced during the resolution phase of inflammation [43,44], and RvD1 has been previously shown to regulate airway inflammation by reducing pro-inflammatory cytokine production and inducing an M2-like macrophage polarization phenotype [27,43,44,45,46]. Our 5 h model of acute dust exposure has been shown to induce an M1-type macrophage phenotype in challenged mice [47], while our NanoString gene expression analyses suggest a potential M2-like polarization of the immune response (e.g., increased *Marco* expression). Considered together with our findings of increased blood DHA levels, elevated BALF RvD1, and increased macrophage influx, these outcomes suggest that the DHA diet is inducing alterations in the kinetics of the inflammatory cascade, possibly promoting the inflammation-resolution response. However, a limitation of our current study is the lack of characterization of the lung macrophage subsets for activation/polarization profiles.

Interestingly, we also identified alterations in neutrophil activities when comparing control-diet-fed with DHA-diet-fed animals. Here, we saw a trend towards reduced neutrophil influx in DHA-diet-fed mice versus control-diet-fed mice exposed to HDE that did not reach statistical significance (*p* = 0.070) yet found enhanced levels of MPO in the BALF of DHA-diet-fed mice and similar levels of NET formation. These data imply that the entering neutrophils may have enhanced antimicrobial activity (based on increased MPO release) in the setting of a high-DHA diet or that infiltrating neutrophils were more quickly subsiding or being efferocytosed by our five-hour timepoint. Together, these findings warrant future studies aimed at identifying the inflammation resolution kinetics following acute HDE exposure, including how PUFAs alter these kinetics, particularly in neutrophils and macrophages.

The gene expression analysis performed in this study details some of the effects a DHA-rich diet can have on immune-associated networks. We found that the main driver for the activation of the lung immune response was the HDE exposure itself, as opposed to dietary intervention (Figure 7). The top 20 significantly altered genes for HDE vs. saline exposure were input into the STRING database and produced a significant interaction (*p* < 1.0 × 10^−16^). Pathway analysis using nSolver and the STRING database showed that most of these 20 genes are involved in a variety of biological processes including cell surface receptor signaling and signal transduction. Among the 20 DHA vs. control diet samples, *ITGAX*, *PIGR*, and *TGFBI* were up-regulated while *CXCL12*, *ITGA6*, and *STAT5B* were down-regulated (Figure 6A). Assessment of the 13 DHA vs. control diet samples with exposure to HDE identified six genes that were significantly upregulated (*BCL3, CFB*, *ITGAX*, *LILRB3*, *MARCO*, and *TGFBI*), while only *STAT5B* was down-regulated (Figure 6B). A list of all relevant genes and their expression/fold changes are presented for DHA-related samples and all other sample groups in Table 3 and Table 4, respectively. The genes listed in Table 4 are expressed among all the analysis sets, whereas *BCL3*, *LILRB3*, and *MARCO* are unique to the DHA-vs.-control-diet-with-HDE analysis (Table 3) because the threshold counts for these three genes were < 82 counts for the saline-related samples.

Transforming growth factor-β-induced (*TGFBI*) is a negative regulator of TLR signaling [48,49], and the increased expression of *TGFBI* has been shown to aid in wound healing and increase macrophage endocytosis [50,51]. Integrin α6 (*ITGA6*) is expressed in the epithelia and forms a complex with either β1 or β4 integrins to function as a receptor for laminin, a basement membrane protein [52]. Additionally, studies have shown that during epithelial injury, the expression of the integrin α6β4 complex increases, suggesting that the decreased expression of *ITGA6* we identified could be due to DHA altering dust-induced inflammation and lung injury [52,53]. Prior research has shown that organic dusts from swine facilities induce CD11c+ macrophages, which can mitigate inflammatory responses in the lung [54,55]. The expression of *ITGAX* (the gene encoding CD11c) in both DHA-diet sample sets suggests that in the setting of a high DHA diet, alveolar macrophages may be playing a role in altering the inflammatory response. This outcome corresponds to the increased BALF macrophages of HDE-treated mice fed a DHA-rich diet and collectively suggests that the resulting increased omega-3 PUFAs may alter the recruitment and/or activation of infiltrating and resident macrophages in the lung following a single dust exposure.

In addition to our findings of elevated BALF macrophage levels and increased CD11c transcripts in the lungs of DHA-diet-fed mice challenged with HDE, we also identified significant alterations in *MARCO* (macrophage receptor with collagenous structure). *MARCO* is known to play a role in M2-like macrophage polarization to aid in host pathogen defense with additional known roles in inflammation resolution and repair processes [56,57]. In conjunction with our findings of increased RvD1 levels, our finding of the increased expression of *MARCO* in our DHA-diet-fed mice further supports the effectiveness of a high-DHA diet in promoting a pro-resolution/repair response following inflammatory insult in the lung. Leukocyte Immunoglobulin Like Receptor B3 (*LILRB3*) also codes for an anti-inflammatory protein expressed on monocytes, dendritic cells, and granulocytes; has been suggested to inhibit stimulated inflammatory responses; and is similarly elevated in DHA-related samples [58,59,60]. Furthermore, we see increased expression of Polymeric Immunoglobulin Receptor (*PIGR*) in our DHA-diet samples; *PIGR* serves as a transporter of IgA across mucosal epithelial cells to help reduce the inflammation stimulated by foreign pathogens [61,62]. The increased expression of this receptor has been shown to occur following the release of the pro-inflammatory cytokines TNF-α and IFN-γ that are produced in response to viral or bacterial infection [61,62]. As previously reported, lipopolysaccharide (LPS) exists within the dusts from swine confinement facilities and contributes to the inflammatory response seen in this exposure model [9,12]. The inhibition of LPS-induced TNF-α expression in macrophages has been shown to be mediated by IL-10, the expression of which is promoted by *BCL3* (B-Cell Leukemia/Lymphoma) [63]. The expression of *BCL3* in our DHA-diet samples is supported by *BCL3′s* ability to promote the downstream inhibition of the LPS-stimulated TNF-α expression in macrophages.

Prior research has shown that elevated levels of complement factor B (*CFB*) are associated with inflammation and airway hyperresponsiveness [64]. The up-regulation of *CFB* was only seen in our DHA-diet samples that were exposed to HDE. Since we only see increased expression of *CFB* in the DHA samples with HDE exposure, it could be suggested that in addition to altering the effects of HDE-induced inflammation, DHA also strengthens overall host immunity to bacterial/viral challenge. In this case, DHA would be functioning to promote systemic immunity to foreign pathogens—a hypothesis that is also supported by the increased levels of MPO release in the DHA-diet- versus control-diet-fed mice treated with HDE. In addition to the various receptors and ECM-related genes differentially expressed in DHA-related samples, we see significant alterations in different cytokine- and chemokine-related genes. The pro-inflammatory chemokine ligand 12 (*CXCL12*) binds to CXCR4 and has previously been shown to recruit numerous cell types such as lymphocytes, monocytes, and eosinophils into the lung airway fluid (BALF) of asthma patients [65]. The decreased expression of *CXCL12* in the DHA- vs. control-diet analysis set suggests that a high-DHA diet modulates the expression of pro-inflammatory responses at the transcriptomic level. Similarly, *STAT5B* has been shown to be activated by IL-5 and GM-CSF activation; *STAT5B* recruits eosinophils into the airways and causes a downstream allergic response [66,67,68]. The down-regulation of *STAT5B* in both DHA-diet-related analysis sets demonstrates the impact of omega-3 PUFAs in reducing the HDE-stimulated inflammatory response.

## 5. Conclusions

In conclusion, a high-DHA diet significantly alters the lung’s inflammatory response to acute agricultural dust exposure, likely by increasing omega-3 PUFAs, which serve as substrates for SPM generation to modify the inflammatory response stimulated by HDE exposure. Evidence in support of this hypothesis includes the elevated blood levels of omega-3 PUFAs, increased macrophage influx into the lung, increased BALF RvD1, and altered gene expression signature associated with macrophage activities in DHA-diet-fed mice exposed to dust compared to mice fed a diet without DHA. These findings warrant future studies to ascertain the potential preventative or therapeutic roles for omega-3 PUFAs in reducing the lung’s inflammatory response to particulate matter exposures such as those experienced in agricultural settings.

## Figures and Tables

**Table 1:** Mean ratio of omega-6 polyunsaturated fatty acids (PUFAs) to omega-3 PUFAs in blood of mice fed a high-DHA diet or control (no DHA) diet for 4 weeks and challenged with saline or HDE.

Experimental Group	Omega-6 PUFA/Omega-3 PUFA Ratio (95% CI)	*p*-Value *
**Control Diet, Saline**	6.20: 1 (3.58:1–8.82:1)	--
**Control Diet, HDE**	7.38:1 (4.91:1–9.85:1)	0.79
**DHA Diet, Saline**	2.34:1 (1.15:1–3.52:1)	0.039
**DHA Diet, HDE**	2.36:1 (1.40:1–3.31:1)	0.021

CI: Confidence interval; * *p*-values based on 2-way ANOVA with Tukey’s multiple comparisons post hoc analysis. Main effect of diet, *p* < 0.0001.

**Table 2:** Breakdown of differential expression for statistically significant genes from each experimental group.

Experimental Group	Number of Samples per Group	Differentially Regulated Genes		Total Number of Differentially Regulated Genes
		*Up*	*Down*	
DHA vs. Control Diet	20	3	3	6
HDE vs. Saline	20	94	16	110
DHA vs. Control Diet Exposed to HDE	13	6	1	7

**Table 3:** Fold regulation of expressed genes in DHA-related samples.

Gene Symbol	Fold Change In Each Data Set	
	DHA vs. Control Diet	DHA vs. Control Diet, Exposed to HDE
*BCL3*	^b^ Below Threshold	↑ 2.24
*CFB*	^a^ ↑ 1.48	↑ 1.57
*CXCL12*	↓ 1.41	^a^ ↓ 1.37
*ITGA6*	↓ 1.52	^a^ ↓ 1.40
*ITGAX*	↑ 1.31	↑ 1.47
*LILRB3*	^b^ Below Threshold	↑ 1.59
*MARCO*	^b^ Below Threshold	↑ 2.07
*PIGR*	↑ 1.44	^a^ ↑ 1.57
*STAT5B*	↓ 1.45	↓ 1.61
*TGFBI*	↑ 1.2	↑ 1.31

^a^ Denotes expression values were not significant (*p* > 0.05). ^b^ Denotes expression was below threshold (< 82 counts); could not assess.

**Table 4:** Fold regulation of expressed genes in diet/exposure groups compared to control diet + saline exposure group.

Gene Symbol	Fold Change/Regulation		
	Control Diet + HDE	DHA Diet + Saline	DHA Diet + HDE
*CFB*	↑ 3.66	^a^ ↑ 1.32	↑ 5.77
*CXCL12*	^a^ ↓ 1.35	^a^ ↓ 1.47	↓ 1.83
*ITGA6*	^a^ ↓ 1.37	^a^ ↓ 1.74	↓ 1.93
*ITGAX*	^a^ ↑ 1.01	^a^ ↑ 1.07	↑ 1.47
*PIGR*	↑ 2.89	^a^ ↑ 1.21	↑ 4.54
*STAT5B*	^a^ ↑ 1.03	^a^ ↓ 1.20	↓ 1.56
*TGFBI*	^a^ ↓ 1.08	^a^ ↑ 1.02	^a^ ↑ 1.06

^a^ Denotes expression values were not significant (*p* > 0.05).

## Supplementary Materials

The following are available online at https://www.mdpi.com/2072-6643/12/8/2334/s1. Figure S1: Initial and Final weights of mice fed a control diet or high DHA diet for 4 weeks.
