# Supplementary material for: A High Docosahexaenoic Acid Diet Alters the Lung Inflammatory Response to Acute Dust Exposure
Source: Nutrients. 2020 Aug 4;12(8):2334. doi: 10.3390/nu12082334 (PMC7468878; doi:10.3390/nu12082334)
Supplement: Supplementary file 1 [file nutrients-12-02334-s001.pdf]

SUPPLEMENTAL DATA

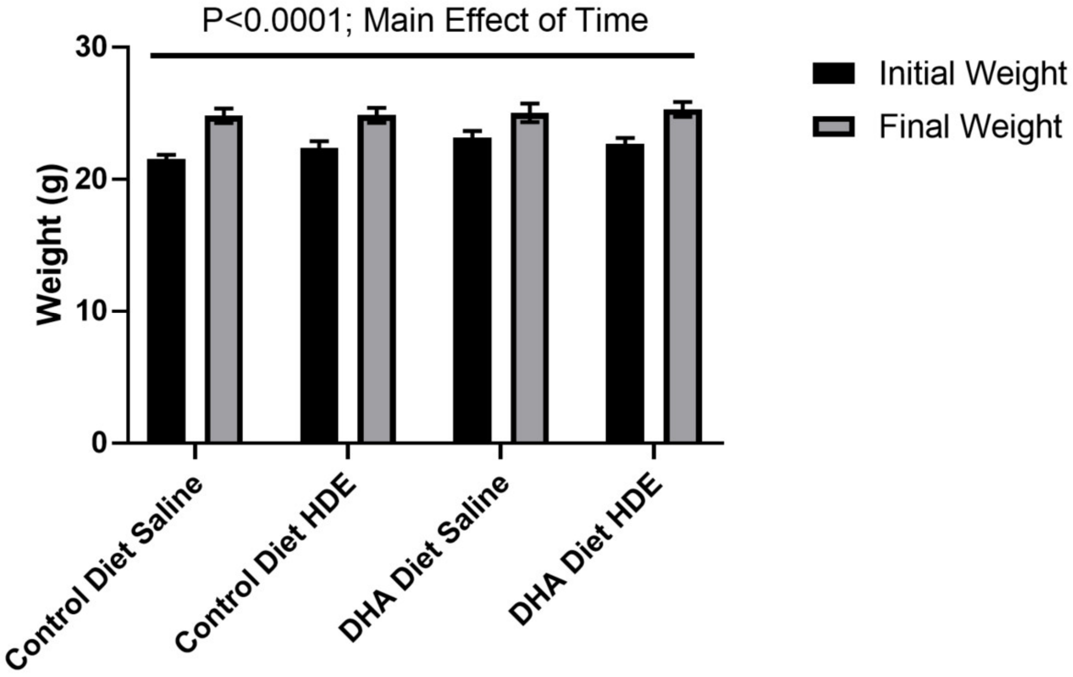

Figure S1. Initial and Final weights of mice fed a control diet or high DHA diet for 4 weeks. A significant main effect of time was identified ($p < 0.0001$), with mice in all experimental groups gaining weight over the 4-week period. There were no significant impacts of diet/HDE treatment on animal weights.
